# Adjuvant Radiation and Endocrine Therapy in Early-Stage Breast Cancer With Low Genomic Risk

**DOI:** 10.1001/jamanetworkopen.2025.32305

**Published:** 2025-09-17

**Authors:** David Gibbes Miller, Lillian A. Boe, Hannah Y. Wen, Boris Mueller, John J. Cuaron, J. Isabelle Choi, Michael B. Bernstein, Beryl McCormick, Simon N. Powell, Atif J. Khan, Lior Z. Braunstein

**Affiliations:** 1 Department of Radiation Oncology, Memorial Sloan Kettering Cancer Center, New York, New York; 2 Department of Epidemiology and Biostatistics, Memorial Sloan Kettering Cancer Center, New York, New York; 3 Department of Pathology, Memorial Sloan Kettering Cancer Center, New York, New York

## Abstract

**Importance:**

De-escalation of adjuvant therapy is feasible for select older patients with early-stage breast cancer. It is not known whether we can expand de-escalation options for younger patients by incorporating genomic biomarkers, such as the Oncotype DX 21-gene recurrence score (ODX RS).

**Objective:**

To evaluate outcomes of radiotherapy (RT) or endocrine therapy (ET) de-escalation for patients aged 50 to 69 years with early-stage breast cancer and an ODX RS of 18 or below.

**Design, Setting, and Participants:**

This cohort study was conducted at a comprehensive cancer center among patients aged 50 to 69 years with T1N0, hormone receptor–positive, *ERBB2*-negative breast cancer, with an ODX RS of 18 or below. Patients were treated between January 2007 and January 2023 with lumpectomy and ET, with or without adjuvant RT. Patients were considered adherent to ET if they received 5 years of ET or more, or if it was ongoing at last follow-up; nonadherence included halting ET within 5 years after initiation.

**Main Outcomes and Measures:**

Cumulative incidence of locoregional recurrence (LRR) was the primary end point, with death and non–local-regional events as competing risks.

**Results:**

This analysis included 2249 patients (median [IQR] age, 60 years [55-65 years]) with a median (IQR) follow-up of 63.3 months (34.1-96.0 months), of whom 2075 (92.3%) received RT. The 72-month cumulative incidence of LRR without RT was 8.0% (95% CI, 3.0%-16.0%) vs 1.1% with RT (95% CI, 0.6%-1.7%) (*P* < .001). When stratified by RT and ET adherence, patients receiving RT had the lowest LRR risk regardless of ET duration (72-month LRR: RT and ET adherence, 1.1% [95% CI, 0.6%-2.1%] vs RT and ET nonadherence, 0.9% [95% CI, 0.3%-2.1%]). Adherence to ET alone (without RT) had an estimated 72-month LRR of 5.5% (95% CI, 1.0%-16.0%). Those who did not receive RT and were ET nonadherent had an estimated 72-month LRR of 11.0% (95% CI, 3.3%-25.0%). No association was observed between receipt of RT and OS (*P* = .2).

**Conclusions and Relevance:**

In this cohort study of patients aged 50 to 69 years who underwent lumpectomy for early-stage breast cancer with ODX RS of 18 or below, we found significantly lower estimated LRR rates among those who completed at least 1 form of adjuvant therapy. For patients willing to accept a low absolute risk, but slightly higher relative risk of LRR with de-escalated therapy, ODX RS may be a valuable tool in selecting younger candidates for RT omission than current guidelines support.

## Key Points

**Question:**

Is omission of adjuvant radiotherapy (RT) or endocrine therapy (ET) associated with higher locoregional recurrence (LRR) rates among patients with early-stage breast cancer and low genomic risk?

**Findings:**

In this cohort study of 2249 patients aged 50 to 69 years with early-stage, hormone receptor–positive breast cancer and an Oncotype DX score of 18 or below, patients receiving RT had the lowest 72-month estimated LRR regardless of ET duration, followed by patients receiving ET alone. Patients who did not complete any adjuvant therapy had the highest LRR.

**Meaning:**

These findings suggest that adjuvant RT or ET can significantly reduce the risk of LRR among patients with early-stage breast cancer and low genomic risk; de-escalated adjuvant therapy may yield acceptable outcomes for such patients, yet shared decision-making remains important as each treatment modality offers its own risks among modest benefits.

## Teaser

This cohort study of US patients with breast cancer examines the association of de-escalating adjuvant therapies with locoregional recurrence and other risks.

## Introduction

Breast conserving therapy (BCT), consisting of lumpectomy and adjuvant radiotherapy (RT), has been a standard treatment option for early-stage breast cancer for several decades.^1,2,3,4^ BCT offers excellent disease control and breast cancer–related mortality outcomes.^2^ Whereas the landmark studies that established a role for RT following lumpectomy were conducted prior to the era of disease subtyping and molecular profiling, the observation of subgroups at very low risk of recurrence has heightened interest in de-escalating adjuvant treatment for select populations.^5,6,7,8,9^ This movement is driven by a desire to maintain excellent outcomes while limiting overtreatment, toxic effects, inconvenience, and cost.

De-escalation of adjuvant therapy may be achieved by omission of RT, which has become acceptable for highly select subgroups of patients with breast cancer. The PRIME II^10^ and CALGB 9343^11,12^ trials both showed that clinicopathologic features can identify very low risk groups of older patients for whom adjuvant RT yields a modest local control benefit without commensurate survival implications. Ongoing studies seek to further identify low-risk groups by incorporating molecular and genomic biomarkers.^13,14^ Recent single-group studies, including the LUMINA,^15^ IDEA,^16^ and PRECISION^17^ trials, have found that features such as Ki67 and genomic recurrence scores can help to identify younger low-risk patients who may also benefit only modestly from RT.

Among informative biomarkers for risk profiling, the Oncotype DX 21-gene recurrence score (ODX RS) has garnered considerable interest. The ODX RS is robustly prognostic of distant recurrence and predictive of chemotherapy benefit, and is thus routinely used for systemic therapy decision-making.^18,19,20^ Emerging data further suggest that the ODX RS correlates with locoregional recurrence (LRR) and, therefore, may be instructive for local therapy decisions.^21,22,23,24,25^ The IDEA trial, for example, identified a subset of patients aged 50 to 69 years with ODX RS of 18 or below who had exceedingly few local failures in the first 5 years following lumpectomy without adjuvant RT, despite variable adherence to endocrine therapy (ET).^16^ Randomized studies on the use of ODX RS and other molecular assays for RT decision-making are in progress, including the DEBRA trial (ClinicalTrials.gov identifier, NCT04852887) and the EXPERT trial (ClinicalTrials.gov identifier, NCT02889874).

Here, we evaluate pragmatic clinical outcomes among a cohort of younger low-risk patients with low ODX RS (ages 50 to 69 years with T1N0, hormone receptor–positive, *ERBB2* [formerly *HER2*]-negative breast cancer and ODX RS 18) who received various permutations of adjuvant RT and ET. We sought to elucidate locoregional recurrence (LRR) and disease-free survival (DFS) rates among this cohort of putatively low-risk patients with and without RT or 5 years of adjuvant ET.

## Methods

The data for this cohort study were gathered from a prospectively maintained institutional database of patients with breast cancer. This study adhered to the Strengthening the Reporting of Observational Studies in Epidemiology (STROBE) reporting guidelines for observational research. The Memorial Sloan Kettering institutional review board approved this study with a waiver for informed consent because it was retrospective research.

### Patient Population

We selected those with early-stage breast cancer treated with BCT at our institution between January 2007 and January 2023. Eligibility criteria included patients aged 50 to 69 years (inclusive) who were diagnosed with estrogen receptor (ER)–positive, progesterone receptor (PR)–positive, and *ERBB2*-negative breast cancer, with a pathologic stage of T1N0. Clinicopathologic and treatment parameters were abstracted from the medical record. All patients had an ODX RS of 18 or below, and all had negative surgical margins. Patients were considered adherent to ET if they received 5 or more years of ET or if it was ongoing at last follow-up. Halting ET within 5 years after initiation was considered nonadherence. Exclusion criteria included prior chemotherapy and bilateral breast cancer.

### Statistical Analysis

The primary outcome was LRR, defined as the time from surgery to breast cancer recurrence in the ipsilateral breast, chest wall, or regional lymph nodes, with distant recurrence, contralateral breast cancer, and death as competing risks. Secondary outcomes were disease-free survival (DFS), defined as the time between date of first breast surgery to date of local, regional, or distant disease recurrence or death due to any cause, as well as time to distant metastases and overall survival (from time of diagnosis). Patients without recurrence or death were censored at the date of last follow-up for both LRR and DFS.

Univariable and multivariable Fine-Gray competing risk regression models, accounting for death and nonlocoregional recurrence as competing risks, were utilized to evaluate the association between radiotherapy and LRR. Variables were selected for inclusion in the multivariable models based on clinical relevance but were limited due to low numbers of events. Cumulative incidence functions were estimated, with the Gray test used to compare differences between groups. A cumulative incidence analysis of LRR by RT and ET use excluded patients whose duration of endocrine therapy use was not known. Analyses were conducted using R version 4.4.1 (R Project for Statistical Computing), with a 2-sided significance threshold of *P* < .05.

## Results

The overall study cohort included 2249 evaluable patients with ODX RS of 18 or below, with a median (IQR) age of 60 years (55-65 years), and a median (IQR) follow-up of 63.3 months (34.1-96.0 months) (Table 1). Of these, 2075 (92.3%) received adjuvant RT, while 174 (7.7%) did not. Among patients who did not receive RT, 53 (30.5%) were aged 50 to 59 years. Moreover, among those who did receive RT, 1263 (60.9%) received whole breast RT with the remainder largely receiving partial breast irradiation. Sentinel lymph node biopsy was nearly universally performed in this cohort (2221 patients [98.8%]). Lymph nodes harboring isolated tumor cells (N0[i+]) were identified in 46 patients (2.0%).

**Table 1.** Patient Characteristics, Overall and by Radiotherapy Receipt

Characteristic	Patients, No. (%)			*P *value^a^
	Overall (n = 2249)	No (n = 174)	Yes (n = 2075)	
Age, median (IQR)	60 (55-65)	63 (58-66)	60 (54-65)	<.001
Age (categorical), y				
50-59	1041 (46.3)	53 (30.5)	988 (47.6)	<.001
≥60	1208 (53.7)	121 (69.5)	1087 (52.4)	
Histology				
Ductal	1905 (84.7)	155 (89.1)	1750 (84.3)	.25
Lobular	338 (15.0)	19 (10.9)	319 (15.4)	
Papillary	6 (0.3)	NA	6 (0.3)	
Laterality				
Left	1110 (49.4)	91 (52.3)	1019 (49.1)	.42
Right	1139 (50.6)	83 (47.7)	1056 (50.9)	
Axillary surgery				
ALN	28 (1.2)	1 (0.6)	27 (1.3)	.72
SLN	2221 (98.8)	173 (99.4)	2048 (99.7)	
Radiotherapy type				
PBI	445 (21.4)	NA	445 (21.4)	NA
Unknown	367 (17.7)	NA	367 (17.7)	
Whole breast	1263 (60.9)	NA	1263 (60.9)	
Overall tumor grade				
I	662 (29.4)	60 (34.5)	602 (29.0)	.03
II	1397 (62.1)	109 (62.6)	1288 (62.1)	
III	138 (6.1)	3 (1.7)	135 (6.5)	
Unknown	52 (2.3)	2 (1.1)	50 (2.4)	
Pathologic T stage				
T1a	153 (6.8)	12 (6.9)	141 (6.8)	>.99
T1b	966 (42.9)	75 (43.1)	891 (42.9)	
T1c	1130 (50.2)	87 (50.0)	1043 (50.3)	
Pathologic N stage				
N0	2203 (98.0)	171 (98.3)	2032 (97.9)	>.99
N0(i+)	46 (2.0)	3 (1.7)	43 (2.1)	
Multifocal	402 (17.9)	13 (7.5)	389 (18.7)	<.001
Hormonal therapy length				
<5 y	687 (30.5)	74 (42.5)	613 (29.5)	.004
≥5 y or Ongoing	1428 (63.5)	89 (51.1)	1339 (64.5)	
Unknown	134 (6.0)	11 (6.3)	123 (5.9)	
Oncotype DX score, median (range)	13.0 (0-18.0)	12.0 (0-18.0)	13.0 (0-18.0)	.35
Oncotype (categorical)				
<10	633 (28.1)	56 (32.2)	577 (27.8)	.22
≥10	1616 (71.9)	118 (67.8)	1498 (72.2)	
Margins				
<1 mm DCIS	98 (4.4)	4 (2.3)	94 (4.5)	.15
<1 mm invasive	68 (3.0)	3 (1.7)	65 (3.1)	
≤2 mm	113 (5.0)	5 (2.9)	108 (5.2)	
Negative	1970 (87.6)	162 (93.1)	1808 (87.1)	

Abbreviations: ALN, axillary lymph node; DCIS, ductal carcinoma in situ; NA, not applicable; PBI, partial breast irradiation; SLN, sentinel lymph node.

^a^ Wilcoxon rank sum test; Pearson χ^2^ test; Fisher exact test.

On univariable analysis, receipt of adjuvant RT and ODX RS were significantly associated with LRR (Table 2). Patients who received RT had a statistically significantly lower risk of LRR (HR, 0.21; 95% CI, 0.08-0.52; *P* < .001). Similarly, higher ODX RS was associated with increased LRR risk (HR, 1.16 per unit increase; 95% CI, 1.05-1.28; *P* = .005). In this cohort of small, favorable tumors, other variables including patient age, tumor laterality, histologic subtype, pathologic T stage, presence of isolated tumor cells in lymph nodes, and multifocality were not significantly associated with LRR in univariable models. On multivariable analysis, RT receipt remained significantly correlated with LRR (HR, 0.21; 95% CI, 0.09-0.52; *P* < .001), even after adjusting for ET duration and ODX RS (eTable 1 in Supplement 1). ODX RS also retained its independent association with LRR risk (HR, 1.16; 95% CI, 1.05-1.28; *P* = .005).

**Table 2.** Univariable Fine-Gray Subdistribution Hazard Competing Risk Regression Model for Locoregional Recurrence With Competing Risks of Death and Other Recurrence

Characteristic	No.	Event No.	HR (95% CI)^a^	*P* value
Radiotherapy				
No	174	6	1 [Reference]	NA
Yes	2075	30	0.21 (0.08-0.52)	<.001
Age	2249	36	0.95 (0.90-1.00)	.07
Age (categorical), y				
50-59	1041	22	1 [Reference]	NA
≥60	1208	14	0.59 (0.30-1.14)	.12
Laterality				
Left	1110	17	1 [Reference]	NA
Right	1139	19	1.14 (0.59-2.18)	.70
Axillary surgery				
ALN	28	2	1 [Reference]	NA
SLN	2221	34	0.32 (0.08-1.32)	.11
Pathologic T stage				
T1a/T1b	1119	13	1 [Reference]	NA
T1c	1130	23	1.79 (0.91-3.54)	.09
Pathologic N stage				
N0	2203	34	1 [Reference]	NA
N0(i+)	46	2	2.08 (0.48-8.97)	.33
Multifocal				
No	1847	27	1 [Reference]	NA
Yes	402	9	1.44 (0.67-3.06)	.35
Hormonal therapy length				
<5 y	687	11	1 [Reference]	NA
≥5 y or Ongoing	1428	23	1.29 (0.60-2.76)	.52
Unknown	134	2	1.55 (0.34-7.04)	.57
Oncotype DX score	2249	36	1.16 (1.05-1.28)	.005
Oncotype (categorical)				
<10	633	5	1 [Reference]	NA
10-18	1616	31	2.34 (0.91-5.99)	.08

Abbreviations: ALN, axillary lymph node; HR, hazard ratio; NA, not applicable; SLN, sentinel lymph node.

^a^ Subdistribution HR.

**Figure 1.**
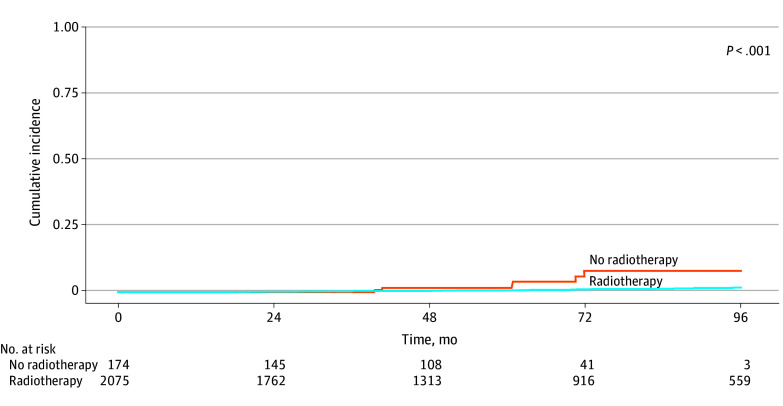
Cumulative Incidence of Locoregional Recurrence by Radiotherapy

**Figure 2.**
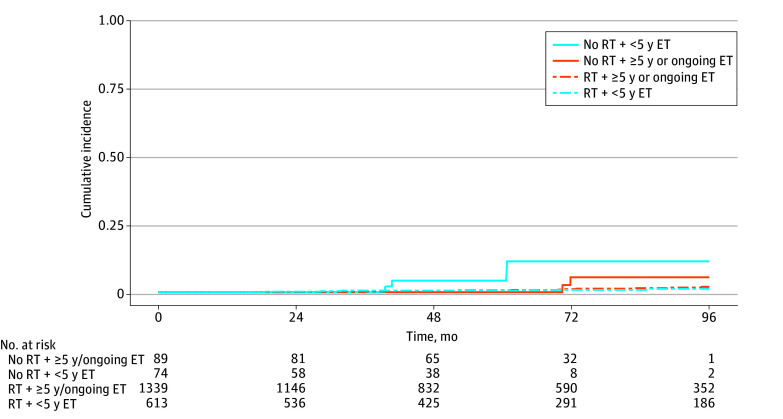
Cumulative Incidence of Locoregional Recurrence, by Radiotherapy (RT) and Endocrine Therapy (ET)

The cumulative incidence of LRR differed significantly by receipt of RT (Figure 1; eTable 2 in Supplement 1): the estimated 72-month LRR was 8.0% (95% CI, 3.0%-16%) for those not receiving RT compared with 1.1% (95% CI, 0.6%-1.7%) with RT (*P* < .001). Moreover, when stratifying by both RT and ET adherence, the lowest estimated incidence of LRR was seen in those who received RT, regardless of ET duration (72-month LRR: RT and 5 years or more [or ongoing] ET, 1.1% [95% CI, 0.6%-2.1%] vs RT and less than 5 years of ET, 0.9% [95% CI, 0.3%-2.1%]) (Table 3). The highest cumulative incidence estimates of LRR were observed among those who did not receive RT and underwent less than 5 years of ET (72-month LRR, 11.0% (95% CI, 3.3%-25.0%). Meanwhile, patients with 5 years or more or ongoing ET (without RT) had higher 72-month LRR estimates than either group that received RT (LRR, 5.5%; 95% CI, 1.0%-16.0%) (Figure 2; eTable 3 in Supplement 1). On univariable Cox regression modeling of DFS, receipt of adjuvant RT was significantly associated with improved DFS (HR, 0.38; 95% CI, 0.19-0.77; *P* = .007) (eTable 4 in Supplement 1). Additionally, patients with 5 or more years or ongoing ET use had a significantly improved DFS as compared with patients receiving fewer than 5 years (HR, 0.53; 95% CI, 0.34-0.82; *P* = .004). Margins containing ductal carcinoma in situ with less than 1 mm clearance were statistically significantly associated with poorer DFS (HR, 2.47; 95% CI, 1.13-5.37; *P* = .02). ODX RS was not significantly associated with DFS. Of note, we identified no association between overall survival and receipt of RT.

**Table 3.** Cumulative Incidence of Locoregional Recurrence at 72 Months, by RT and ET Adherence

Treatment	72-mo Cumulative incidence, % (95% CI)	*P* value^a^
No RT plus ET nonadherent	11.0 (3.3-25.0)	<.001
No RT plus ET adherent	5.5 (1.0-16.0)	
RT plus ET nonadherent	0.9 (0.3-2.1)	
RT plus ET adherent	1.1 (0.6-2.1)	

Abbreviations: ET, endocrine therapy; RT, radiotherapy.

^a^ Gray test; *P* value reflects differences across entire follow-up period.

## Discussion

In this cohort study, we found that among patients 50 to 69 years of age with early-stage breast cancer and ODX RS of 18 or lower, oncologic outcomes are improved with adjuvant therapy. At 72 months, estimated LRR risk following RT was about 1% regardless of ET adherence, increasing to 5.5% without RT but with ET adherence, and to 11% without RT and less than 5 years of ET. These findings demonstrate the effectiveness of adjuvant therapy, particularly RT, even among such low-risk cohorts. These findings also support the potential safety of adjuvant therapy de-escalation through RT or ET omission for patients with breast cancer who are young and with early-stage, low genomic–risk disease, who ordinarily would not be candidates for treatment de-escalation. These patients nevertheless might be willing to accept a slightly higher relative risk of LRR in exchange for de-escalated therapy.

Since early landmark trials cemented a role for adjuvant RT following breast conserving surgery,^26,27^ several efforts have sought to identify favorable subgroups who might not benefit from RT.^3,28^ In contemporary practice, the CALGB 9343 and PRIME II trials demonstrated that older patients with hormone receptor-positive breast cancer may feasibly forgo RT without impinging on survival outcomes despite marginally higher local recurrence risk.^10,11,12^ Many patients older than 65 years now routinely accept this modestly increased risk of LRR in exchange for omission of RT, although supportive data for younger populations have been lacking. Of note, our risk estimates with and without RT are consistent with the outcomes described in the PRIME II and CALGB 9343 trials, which both included variable degrees of adherence to ET. The PRIME II study of patients ages 65 years and older found a 10-year local recurrence risk of 9.5% in the no-RT group vs 0.9% in the RT group,^10^ whereas in the CALGB 9343 trial among those 70 years or older, 10% of those not receiving RT had an LRR vs 2% among those receiving RT.^11,12^

Contemporary trials are now investigating RT omission for younger patients by incorporating transcriptional profiling to identify a favorable-risk subgroup that may not stand to benefit appreciably from aggressive adjuvant therapy. A number of studies have demonstrated that ODX RS is associated with local outcomes,^21,22,23,24,25^ and several nascent trials seek to employ molecular risk stratification for RT decision-making. The LUMINA trial, for example, studied omission of radiation in patients ages 55 years and older with T1N0 breast cancer and luminal A subtype (defined on trial as: ER 1% or more, PR higher than 20%, *ERBB2* negative, with Ki67 of 13.25% or lower), who were treated with adjuvant endocrine therapy alone after lumpectomy. At 5 years, the rates of local recurrence were exceedingly low at 2.3%.^15^ Similarly, the phase 2 IDEA trial was a single-group study among low-risk postmenopausal women with ODX RS of 18 or lower and found a 5-year risk of local recurrence below 4%.^16^ Other ongoing prospective trials evaluating omission of RT include the randomized phase 3 DEBRA trial (ClinicalTrials.gov identifier, NCT04852887),^29^ PRECISION,^17^ and the EXPERT trial,^30^ all of which will serve to elucidate further the role of molecular profiling for RT candidacy.

In the context of the ongoing studies evaluating the implications of RT omission in a younger population,^29^ this analysis provides a pragmatic assessment of clinical outcomes for younger, early-stage, low genomic–risk patients with breast cancer who would have met eligibility criteria for the IDEA or DEBRA trials. The relative benefits of adjuvant therapy in this population were clear, as any adjuvant therapy demonstrated a significant LRR relative risk reduction. The 11% estimated 72-month LRR among patients who did not receive RT or adhere to ET reinforces the accepted consensus that even this low-risk group of patients stands to benefit from some form of adjuvant therapy. While the addition of adjuvant RT was associated with the lowest 72-month LRR, patients who were adherent to ET and omitted RT also had a very low absolute rate of LRR. Similar to interpretations of the CALGB 9343 and PRIME II studies, these results suggest that it may be reasonable for younger, low-risk patients to forgo adjuvant RT and accept a slightly higher local recurrence risk.

The lowest estimated LRR was seen in patients who received RT, regardless of ET adherence, raising the possibility that ET omission in favor of RT monotherapy may be an acceptable option in this population. Early reports from the EUROPA trial, which is evaluating RT vs endocrine monotherapy for early-stage disease among those age 70 years and older, demonstrate excellent outcomes with either approach, but show that RT may better preserve quality of life than ET.^31^ Our study similarly showed excellent outcomes among those who received adjuvant RT and less than 5 years of ET, lending further support for this reasonable monotherapeutic course. Ultimately, the long-term safety of omitting ET, particularly in terms of distant recurrence and contralateral breast cancer risk, remains an open question to be further evaluated by prospective trials.

Our cohort also predates the publication of the SOUND trial, which demonstrated very low rates of LRR in patients with early-stage, clinically node-negative breast cancer, regardless of receipt of axillary nodal staging. While adjuvant therapy de-escalation may be safely pursued for those who do not undergo pathologic axillary staging, our study only included those who underwent axillary surgery as was the standard until very recently. As omission of surgery axillary evaluation becomes more widely adopted, further research will be needed to understand whether patients without pathologic nodal staging ought to be counseled differently on adjuvant therapy omission.

Our results add to a growing body of evidence supporting RT omission among a younger group of carefully selected early-stage breast cancer patients. They also align with early research supporting the option of adjuvant RT monotherapy and omission of ET for some early-stage breast cancer patients.^32,33,34,35^ Our results show that a variety of adjuvant therapy options are associated with acceptably low LRR, and younger patients with low genomic risk breast cancer may reasonably consider de-escalated adjuvant therapy.

We note that this analysis focused largely on LRR, and our event rate following RT was exceedingly favorable. Going forward, the ability to safely moderate the complexity of adjuvant therapy may provide meaningful quality-of-life benefits while preserving excellent long-term disease control. Longer follow-up and the maturation of related studies will further inform the optimal treatment approach. Ultimately, the results may aid in further informing patients about the relative benefits of different forms of adjuvant therapy, thereby supporting personalized adjuvant therapy decision-making among a carefully selected group of younger patients. These results thus provide pragmatic, clinical data that may empower patients to make informed decisions through shared decision-making, aligning treatment plans with their preferences and values. Ongoing trials and maturing reports will define the feasibility of RT or endocrine monotherapy in this younger population.

### Limitations

This study had several limitations. Our findings should be interpreted in the context of patient selection. Patients who did not receive RT were more likely to be older, have grade I tumors, and have unifocal disease. ET nonadherence was also more prevalent in the no-RT group. Omission of adjuvant therapy was not standard of care for most patients during the study period. As such, patients who declined RT or were nonadherent to ET likely self-selected this treatment course. It is plausible that younger patients or those with higher-grade or multifocal tumors were more strongly advised to pursue full adjuvant therapy, potentially accounting for the observed differences between groups. It is also possible that patients who declined RT were similarly predisposed to forgo ET as well. Taken together, these subtle imbalances, if anything, reinforce the effect of RT given that patients receiving RT had slightly less favorable clinicopathologic profiles.

As a retrospective analysis, our data were susceptible to bias and confounding, although we attempted to mitigate these issues by presenting multivariable analyses and fully characterizing the clinicopathologic features among both the RT and no-RT cohorts. Despite the generous sample size and long follow-up, the low-risk nature of this population yielded a necessarily low event rate, limiting our ability to perform more extensive multivariable analyses. Ongoing prospective studies will help to elucidate any potential residual confounding between the clinicopathologic features studied herein and disease outcomes.

## Conclusions

In this cohort study of patients aged 50 to 69 years who underwent lumpectomy for early-stage breast cancer with ODX RS of 18 or lower, we found very low estimated LRR rates among patients who received adjuvant therapy. The LRR rates remained low among patients who omitted adjuvant RT or were ET nonadherent.

## Notes

Supplement 1. **eTable 1.** Multivariable Fine-Gray Subdistribution Hazard Regression Model for Locoregional Recurrence with Competing Risks of Death and Other Recurrence **eTable 2.** Cumulative Incidence of Locoregional Recurrence with Competing Risk of Death and Other Recurrence, by Radiotherapy Receipt **eTable 3.** Cumulative Incidence of Locoregional Recurrence with Competing Risk of Death and Other Recurrence, by Radiotherapy Receipt and Endocrine Therapy Adherence **eTable 4.** Univariable Cox Proportional Hazards Regression Models for Disease-Free Survival

Supplement 2.
Data Sharing Statement
