# Supplementary material for: Adjuvant Radiation and Endocrine Therapy in Early-Stage Breast Cancer With Low Genomic Risk
Source: JAMA Netw Open. 2025 Sep 17;8(9):e2532305. doi: 10.1001/jamanetworkopen.2025.32305 (PMC12444576; doi:10.1001/jamanetworkopen.2025.32305)
Supplement: Supplement 1. — eTable 1. Multivariable Fine-Gray Subdistribution Hazard Regression Model for Locoregional Recurrence with Competing Risks of Death and Other Recurrence eTable 2. Cumulative Incidence of Locoregional Recurrence with Competing Risk of Death and Other Recurrence, by Radiotherapy Receipt eTable 3. Cumulative Incidence of Locoregional Recurrence with Competing Risk of Death and Other Recurrence, by Radiotherapy Receipt and Endocrine Therapy Adherence eTable 4. Univariable Cox Proportional Hazards Regression Models for Disease-Free Survival [file jamanetwopen-e2532305-s001.pdf]

Supplemental Online Content

Miller DG, Boe LA, Wen HY, et al. Optimizing adjuvant radiation and endocrine therapy in early-stage breast cancer with low genomic risk. *JAMA Netw Open.* 2025;8(9):e2532305. doi:10.1001/jamanetworkopen.2025.32305

eTable 1. Multivariable Fine-Gray Subdistribution Hazard Regression Model for Locoregional Recurrence with Competing Risks of Death and Other Recurrence

eTable 2. Cumulative Incidence of Locoregional Recurrence with Competing Risk of Death and Other Recurrence, by Radiotherapy Receipt

eTable 3. Cumulative Incidence of Locoregional Recurrence with Competing Risk of Death and Other Recurrence, by Radiotherapy Receipt and Endocrine Therapy Adherence

eTable 4. Univariable Cox Proportional Hazards Regression Models for Disease-Free Survival

This supplemental material has been provided by the authors to give readers additional information about their work.

eTable 1. Multivariable Fine-Gray Subdistribution Hazard Regression Model for Locoregional Recurrence with Competing Risks of Death and Other Recurrence

Characteristic	N	Event N	HR[a] (95% CI)	p-value
Radiotherapy				
No	174	6	N/A	
Yes	2,075	30	0.21 (0.09-0.52)	<0.001
Hormone Therapy Length				
<5 years	687	11	N/A	
5+ years/ongoing	1,428	23	1.29 (0.60-2.76)	0.5
Unknown	134	2	1.68 (0.36-7.78)	0.5
Oncotype DX Score	2,249	36	1.16 (1.05-1.28)	0.005

[a]Subdistribution hazard ratio

© 2025 Miller DG et al. *JAMA Network Open*.

eTable 2: Cumulative Incidence of Locoregional Recurrence with Competing Risk of Death and Other Recurrence, by Radiotherapy Receipt

Characteristic	24-month (95% CI)	48-month (95% CI)	72-month (95% CI)	96-month (95% CI)	p-value[1]
Radiotherapy					<0.001
No	0.00% (N/A)	1.6% (0.31-5.1)	8.0% (3.0-16)	8.0% (3.0-16)	
Yes	0.16% (0.05- 0.46)	0.42% (0.19-0.84)	1.1% (0.59-1.7)	1.7% (1.0-2.7)	

[1]Gray's Test

© 2025 Miller DG et al. *JAMA Network Open*.

eTable 3. Cumulative Incidence of Locoregional Recurrence with Competing Risk of Death and Other Recurrence, by Radiotherapy Receipt and Endocrine Therapy Adherence

Characteristic	24-month (95% CI)	48-month (95% CI)	72-month (95% CI)	96-month (95% CI)	p-value[1]
RT/Endocrine Therapy					<0.001
No RT + Endocrine Therapy <5 years	0.00% N/A)	4.3% (0.76-13)	11% (3.3-25)	11% (3.3-25)	
No RT + Endocrine Therapy 5+ years/ongoing	0.00% (N/A)	0.00% (N/A)	5.5% (0.96-16)	5.5% (0.96-16)	
RT + Endocrine Therapy <5 years	0.18% (0.02-0.95)	0.57% (0.16-1.6)	0.85% (0.28-2.1)	1.3% (0.45-2.9)	
RT + Endocrine Therapy 5+ years/ongoing	0.09% (0.01-0.48)	0.29% (0.08-0.82)	1.1% (0.55-2.1)	2.0% (1.1-3.4)	

[1]Gray's Test

© 2025 Miller DG et al. *JAMA Network Open*.

eTable 4. Univariable Cox Proportional Hazards Regression Models for Disease-Free Survival

Characteristic	N	Event N	HR (95% CI)	p-value
**Radiotherapy**				
No	174	9	N/A	
Yes	2,075	80	0.38 (0.19-0.77)	0.007
**Age**	2,249	89	1.02 (0.99-1.06)	0.2
**Age (Categorical)**				
50-59	1,041	37	N/A	
60+	1,208	52	1.32 (0.87-2.02)	0.2
**Laterality**				
Left	1,110	48	N/A	
Right	1,139	41	0.86 (0.57-1.31)	0.5
**Axillary Surgery**				
ALN	28	3	N/A	
SLN	2,221	86	0.52 (0.17-1.66)	0.3
**Pathologic T Stage**				
T1a	153	3	N/A	
T1b	966	29	1.74 (0.53-5.73)	0.4
T1c	1,130	57	3.01 (0.94-9.62)	0.063
**Pathologic N Stage**				
N0	2,203	87	N/A	
N0(i+)	46	2	0.80 (0.20-3.27)	0.8
**Multifocal**				
No	1,847	68	N/A	
Yes	402	21	1.32 (0.81-2.15)	0.3
**Hormonal Therapy Length**				
<5 years	687	42	N/A	
5+ years/ongoing	1,428	39	0.53 (0.34-0.82)	0.004
Unknown	134	8	1.60 (0.75-3.42)	0.2
**Oncotype DX Score**	2,249	89	1.03 (0.98-1.09)	0.2
**Oncotype (Categorical)**				
<10	633	24	N/A	
10+	1,616	65	1.01 (0.63-1.61)	>0.9
**Margins**				
Negative	1,970	75	N/A	
< 1mm DCIS	98	7	2.47 (1.13-5.37)	0.023
< 1mm Invasive	68	4	1.73 (0.63-4.74)	0.3
<=2 mm	113	3	0.57 (0.18-1.82)	0.3

© 2025 Miller DG et al. *JAMA Network Open*.
