# Supplementary material for: Adjuvant Radiation and Endocrine Therapy in Early-Stage Breast Cancer With Low Genomic Risk
Source: JAMA Netw Open. 2025 Sep 17;8(9):e2532305. doi: 10.1001/jamanetworkopen.2025.32305 (PMC12444576; doi:10.1001/jamanetworkopen.2025.32305)
Supplement: Supplement 2. — Data Sharing Statement [file jamanetwopen-e2532305-s002.pdf]

Data Sharing Statement

Miller. Optimizing Adjuvant Radiation and Endocrine Therapy in Early-Stage Breast Cancer with Low Genomic Risk. *JAMA Netw Open*. Published September 17, 2025. doi:10.1001/jamanetworkopen.2025.32305

Data
Data available: No
